# Patient perspectives on health‐related behavior change after transient ischemic attack or ischemic stroke

**DOI:** 10.1002/brb3.1993

**Published:** 2021-03-04

**Authors:** Dorien Brouwer‐Goossensen, Heleen M. den Hertog, Marinke A. Mastenbroek‐de Jong, Lisette J. E. W. C. van Gemert‐Pijnen, Erik Taal

**Affiliations:** ^1^ Erasmus MC University Medical Center Rotterdam Rotterdam The Netherlands; ^2^ Isala Hospital Zwolle The Netherlands; ^3^ University of Twente Twente The Netherlands

**Keywords:** health‐related behavior, patient perspectives, search terms: stroke, TIA

## Abstract

**Objective:**

Unhealthy lifestyle is common among patients with ischemic stroke or TIA. Hence, health‐related behavior change may be an effective way to reduce stroke recurrence. However, this is often difficult to carry out successfully. We aimed to explore patients' perspectives on health‐related behavior change, support in this change, and sustain healthy behavior.

**Methods:**

We conducted a descriptive qualitative study with in‐depth, semistructured interviews in eighteen patients with recent TIA or ischemic stroke. Interviews addressed barriers, facilitators, knowledge, and support of health‐related behavior change framed by the protection motivation theory. All interviews were transcribed and thematically analyzed.

**Results:**

Patients seem unable to adequately appraise their own health‐related behavior. More than half of the patients were satisfied with their lifestyle and felt no urgency to change. Self‐efficacy as coping factor was the most important determinant (both barrier and facilitator). Fear as threat factor was named as facilitator for health‐related behavior change by half of the patients. Most of the patients did not need support or already received support in changing health behavior. Patients indicated knowledge, guidelines, and social support as most needed to support and preserve a healthy lifestyle.

**Conclusion:**

This study suggests that patients with recent TIA or ischemic stroke often do not have a high intention to change health‐related behavior. The results fit well within the framework of the protection motivation theory. As many patients seem unable to adequately appraise their health behaviors, interventions should focus on increasing knowledge of healthy behavior and improving self‐efficacy and social support.

## Graphical abstract

TIA and stroke patients understand what constitutes a healthy lifestyle, but seem unable to adequately appraise their own health‐related behavior. More than half of the patients were satisfied with their lifestyle and felt no urgency to change. Self‐efficacy was the most important determinant for health‐related behavior change and mentioned both as barrier and as facilitator.

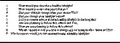

## 1 BACKGROUND

Stroke is the third cause of death and the first cause of disability in developed countries (Lozano et al., 2012). Transient ischemic attacks (TIAs) can be seen as a warning sign and require urgent evaluation to prevent a stroke (Easton et al., 2009). As recurrence rates are high (Hankey et al., 2000), management of risk factor and health behavior is of great importance. Interventions promoting a healthy lifestyle after TIA or ischemic stroke may be an effective way to reduce stroke recurrence and are strongly recommended in many guidelines (European Stroke Initiative Executive C et al., 2003; Kernan et al., 2014; Rudd et al., 2017). Recommended lifestyle behaviors to prevent recurrence after TIA or ischemic stroke include regular physical exercise (more than 30 min of moderate or intense activity a day), healthy diet, stop smoking, and no excessive use of alcohol. However at present, only limited and inconsistent data are available on interventions to support patients in health‐related behavior change after TIA or ischemic stroke (Ellis et al., 2005; Lawrence et al., 2010; Lennon, Galvin, et al., 2013a; Maasland et al., 2007; Rodgers et al., 1999; Sit et al., 2007).

Health‐related lifestyle change after ischemic stroke and TIA is difficult to carry out successfully, and the majority of people fail to sustain lifestyle modification in the long term (Allison et al., 2008; Redfern et al., 2000). Patients' knowledge about risk factors for ischemic stroke or TIA is often poor (Croquelois & Bogousslavsky, 2006), and even when patients believe that their lifestyle is related to their stroke, they do not change their smoking or excessive alcohol drinking habits (Yuki & Kudo, 2011). Patients experience physical barriers such as pain, fatigue balance problems, or fear of falling. Reported mental barriers include lack of motivation or social support and boredom, which contributed to persistent smoking. Also, environmental barriers such as bad weather, bad roads, and costs of healthy foods were experienced as barriers for behavior change (Lennon, Doody, et al., 2013).

**Figure 1:**
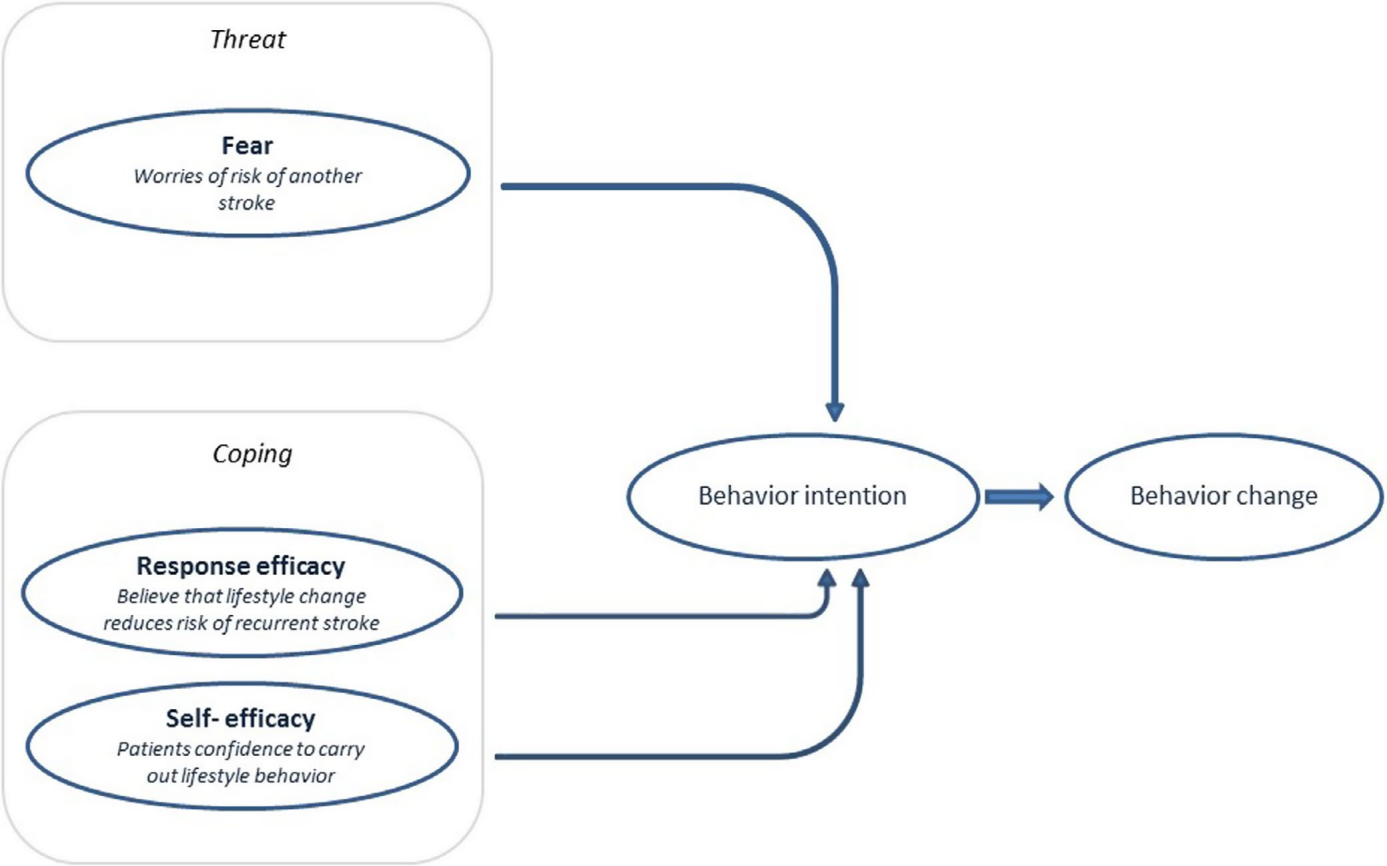
Used protection motivation theory factors

The process of behavior change is complex and has been described in several models. Roger's revised protection motivation theory (PMT) (Rogers, 1975) describes cognitive factors that play a role in individual's motivation to change or not to change health‐related behavior. Similar to other models, this theory assumes that behavior change is a consequence of behavioral intention to change. An intention to change only develops when a threat is perceived and a coping response is available. Fear of a recurrent stroke is often present after stroke or TIA. According to the protection motivation theory, this fear motivates patients to make changes in their lifestyle to promote their health in order to avoid a new stroke if patients are confident they are able to carry out these lifestyle behaviors (Bendz, 2003; Carlsson et al., 2009; Horne et al., 2014; Martin et al., 2002; Nordin et al., 2015; Townend et al., 2006) (Figure 1). In a previous study, we showed that fear of recurrence, self‐efficacy (patients' confidence to carry out lifestyle behavior), and response efficacy (believe that lifestyle behavior change reduces risk of recurrent ischemic stroke) are determinants of intention to change health behavior after TIA or ischemic stroke (Brouwer‐Goossensen et al., 2016). Understanding of patients' perspectives of these determinants of health‐related behavior change after TIA or ischemic stroke can facilitate the development of successful behavior change strategies.

At present, it is unclear how patients judge their own lifestyle after TIA or ischemic stroke, which facilitating factors and barriers for health‐related behavior change are experienced, and which support patients desire to support health‐related behavior change. Hence, we explored patients' perspectives on health‐related behavior change and support in health‐related behavior change after TIA or minor ischemic stroke in a qualitative study with in‐depth, semistructured interviews.

## 2 METHODS

We conducted a descriptive qualitative study with in‐depth, semistructured interviews. Patients were eligible for inclusion if they were 18 years or older and had a clinical diagnosis of TIA or minor ischemic stroke and a modified Rankin Scale score of 3 or less. The modified Rankin Scale (mRS) is a commonly used scale for measuring the degree of disability or dependence in the daily activities of people who have suffered a stroke. Scores on the mRS range from 0 (no symptoms at all) to 5 (severe disability) (Swieten et al., 1988). Eighteen patients with TIA or ischemic stroke were interviewed in the first month after their TIA or ischemic stroke. This is chosen because earlier research showed that fear significantly decreased after 3 months and there seems to be a window of opportunity in changing health‐related behavior shortly after an event (Brouwer‐Goossensen et al., 2021). We included TIA and stroke patients because they often experience the same symptoms, such as cognitive problems, fatigue, and anxiety after their event (Soros et al., 2015) and usually receive the same treatment and outpatient follow‐up. All patients received verbal routine general lifestyle advice including regular physical exercise, healthy diet, and advice to stop smoking as part of standard care of the neurologist. Knowledge about the disease, results of the examinations, and risk factors were discussed with the patient to form a basis for behavioral change and improved awareness of personal risk behavior as this is supposed to be especially important to proceed behavior change (Ronda et al., 2001). In this conversation fear, response efficacy and self‐efficacy were addressed by discussing the risk of recurrence, the effect of behavior change, and possibilities for health behavior change.

We recorded data on quantification of stroke severity according to the National Institutes of Health Stroke Scale (NIHSS) (Schlegel et al., 2003), demographic data, education, and BMI. The trial was approved by national and local institutional review boards, and written informed consent was obtained from all patients.

### 2.1 Interviews

All 18 patients underwent in‐depth interviews of 60 min taken by MdJ. Seventeen patients were interviewed at home and one in the hospital. Interviews were audiotaped, transcribed, and thematically analyzed by MdJ and DBG. Interviews followed a scheme that addressed patients' assessment of their own lifestyle, barriers, and facilitators of health‐related behavior change framed by the protection motivation theory and desired support in the behavior change process (Table 1). Patients were asked to describe a healthy lifestyle and to compare it with their own lifestyle, in order to get information about the knowledge of healthy behavior of these patients. After that patients were asked whether they had changed their lifestyle after the TIA or ischemic stroke and which barriers and facilitating factors they experienced. Questions were asked about the lifestyle factors of smoking, exercise, (healthy) diet and alcohol consumption, and the motivation to change or not to change the lifestyle. Finally, they were asked what type of support they need when changing their lifestyle and what could help to maintain a healthy lifestyle.

**Table 1:** Interview guide

1. How would you describe a healthy lifestyle?
2. How would you describe your lifestyle?
3. Did your lifestyle change after your stroke/TIA?
4. Did you change your lifestyle before?
5. Did you receive advice about a healthy lifestyle in the hospital?
6. Are you planning to follow this advice and if so,
7. How are you planning to follow this advice?
8. Which support would you like in changing your lifestyle after stroke or TIA?
9. Which support would you like in maintaining a healthy lifestyle?

### 2.2 Qualitative analysis

All interviews were analyzed with open, axial, and selective coding using a framework approach (Gale et al., 2013) by the interviewer (MdJ) and researchers (DBG and ET). In the first stage, MdJ listened the interview recordings while reading the transcripts. In the second stage, MdJ divided interviews into fragments, which were classified over the determinants of the protection motivation theory. No qualitative software was used. In the third stage, DBG and ET reviewed this classification. Interviews were read again and fragments further refined. Barriers and facilitators per factors were selected by MdJ and reviewed by DBG and ET.

## 3 RESULTS

Of the eighteen interviewed patients, the mean age was 65 years (IQR 48‐80), 11 (61%) were male, and 14 (78%) had a TIA (Table 2). Most patients had a mRS score of 0 or 1, which means that they were mildly or not disabled. None of the patients used more alcohol than advised, and 3 (17%) of the patients were smokers. Three patients changed their alcohol consumption, and two patients stopped smoking after their TIA of ischemic stroke.

**Table 2:** Baseline characteristics (*n* = 18)

Sex (male), *n* (%)	11 (60)
Age (years), mean (*SD*)	67 (9)
Event characteristics	
Event type (stroke), *n* (%)	14 (78)
NIHSS score^a^, median (IQ)	2 (1,5–3)
Modified Rankin Scale^b^, median (IQ)	1 (0–2)
Education	
8–15 years, *n* (%)	8 (44)
15–17 years, *n* (%)	4 (22)
17–20 years, *n* (%)	6 (33)
Lifestyle	
Smoking, *n* (%)	5 (28)
Alcohol abuse, *n* (%)	4 (22)
BMI (kg/m^2^), mean (*SD*)	28 (4.4)
Overweight (BMI > 25), *n* (%)	7 (47)

^a^ Quantification of stroke severity according to the National Institutes of Health stroke scale, a 15‐item scale with scores that range from 0 to 42 and higher values indicating greater severity.

^b^ Quantification of the degree of disability or dependence in the daily activities of people who have suffered a stroke. Scores on the mRS range from 0 (no symptoms at all) to 5 (severe disability).

### 3.1 Healthy lifestyle perspectives

All patients mentioned a good diet as positive and smoking as negative in relation to a healthy lifestyle.

Just a normal life, quit smoking, reducing alcohol and a healthy lifestyle

Most participants named exercise as part of a healthy lifestyle, and alcohol intake as unhealthy.

A lot of exercise, healthy eating. And smoking is not a part of this and not drinking.

Patients reported that working in their garden, walking with the dog, or walking stairs at work was enough exercise during the day.

Movement, yes I have enough. I walk up and down the stairs all day and go to the studio.

More than half of the patients were satisfied with their own lifestyle. One third of the patients rated their dietary pattern as good, and felt no need to change.

No complaints about my lifestyle. Because I feel pretty good now, why would I change things.

### 3.2 General barriers and facilitators

Five patients named lack of knowledge as a barrier for behavior change, in particular in relation to dietary behavior.

And furthermore they just let me find out … they just let me figure it all out for myself, they do not say what you can do best.

Social support was experienced as a facilitator of physical activity. Support of spouses was named by three patients.

Yes, I do that with my husband …that's really nice… I feel his support, like: together we can do this. So that's really nice.

Some patients appear to have a low perceived severity of their ischemic stroke, which leads to the absence of an intention to quit smoking:

I simply hate it, but I also hate that nothing comes out of those investigations. And therefore I say, well if there is anything that they see, something in my brains, well if there is a bit of a scar, they can see something, then I'm like: shit. But now I just haven't yet.

However, for one participant severity appeared to be a facilitating factor to quit excessive alcohol intake. According to this patient, it was a choice between drinking and dying or quit drinking and stay alive. Severity has not been mentioned in relation to other health‐related behavior.

### 3.3 Self‐efficacy

Self‐efficacy was most common mentioned as a barrier or facilitator of health‐related behavior change.

Self‐confidence I need to have again … Yes, I want to quit, but I can't. I can't.

Mental, physical and environmental barriers were barriers for health‐related behavior change. Mental barriers were mainly mentioned in relation to smoking habits.

Because I feel so much stress. And then I think, now that I had this, this year sucks. … if I have to quit now, I don't have anything left, I feel a bit like that.

Mood and cognitive problems were also experienced as a mental barrier for health‐related behavior change in four patients.

When I am a bit depressed, yes I smoke a bit more, but well then, then I think something like ten cigarettes a day.

Physical complaints, such as pain and fatigue, were remarked as most experienced barrier for physical activity.

I do something and then I am tired and then I sit down again, I am tired very quickly, that is the difference. I am tired very quickly. Since I've had this, yes.

Also, environmental barriers were mentioned. Bad weather (cold or rain) was mentioned as a barrier for physical activity.

Oh, I can't stand the cold. Because of the blood pressure and vessels. So I stay at home.

One patient found it hard to eat healthy because of bad eating habits of her partner.

### 3.4 Response efficacy

Response efficacy was mentioned in relation to all types of health‐related behavior change. Patients experienced response efficacy mostly as facilitating factor for behavior change.

If I can keep up with that …, then my chances of getting another stroke are just as high as any other.

Response efficacy was especially mentioned in relation to changing eating habits and alcohol use.

That's because of the cholesterol, it was too high in my opinion. Because of junk food. So I ate less of that.

Low response efficacy was remarked as a barrier for changing physical activity behavior in six patients. Four patients did not believe that quitting smoking would help.

Yeah, what I had, has nothing to do with smoking (partner: but with vessels and other things) well my vessels are OK, the doctor said. But now, I'm appeasing myself, but okay.

### 3.5 Fear

Nine patients regarded fear of recurrence a facilitating factor for behavior change in smoking, physical activity, and dietary behavior. One patient mentioned that by seeing other (worse) stroke patients, she developed the motivation to change her health‐related behavior.

maybe that was ‘the light’, . That people who were lying next to me couldn't speak well and then, well let's say I'm lucky. But you'll think about it and then you'll go change something, a different lifestyle.

When asking which factors played a role in quitting smoking and reducing alcohol use, one patient found fear an important factor.

Yes… which factors… fear (partner: fear of recurrence, you start thinking about it… because it went well this time but…)

Also, one patient found the ischemic stroke was a wake‐up call and knew that it could happen again. Another patient tried to eat healthier because of fear of recurrence. Fear has also been mentioned as a facilitating factor by a participant who stopped smoking in the past because of fear of lung diseases. Two other participants were not aware of the risk of smoking on TIA or ischemic strokes and did not feel fear of recurrence by keeping smoking.

Yes well I had this, but stop smoking: no. Maybe as a second MRI shows something, that I think: oh.

### 3.6 Hospital advices

Most patients received advices on health‐related behavior during their stay in the hospital or visit to the outpatient clinic. This consisted according to the patients of the following advice: take rest, eat healthy, stay in condition, and use their medication. Some patients did not receive any advice because they did not consider and think it necessary.

Yeah it's all very logical. When you just read something here and there about physical activity, don't smoke, drink less alcohol, so I think, half of that I already do.

More than half of the patients did not need any support to change their health‐related behavior. They mentioned that they had enough knowledge of what was right because of the hospital advice or their own knowledge.

No, I already know. I know enough and when I don't, I will find out myself

Most patients who quit smoking in the past did this without support. Patients also learned about healthy lifestyle because of relatives who received advices.

### 3.7 Support needs

Some patients needed more information about what they can do themselves to prevent another TIA or ischemic stroke. In addition, there was also a need for guidelines on what is and what is not allowed. In particular, advices on healthy diet would be helpful according to three patients.

That you get a little more guidance. Whether it is from the specialist or from the doctor, is not important, only that you have a little more guidance, this is good and this is not good.

Two patients would like support, but had no idea which support could be effective in relation to stop smoking and increasing physical activity.

I would not know. I cannot imagine anything at all.

Patients would like to have professional support in changing behavior and healthy lifestyle preservation mainly in improving physical activity. Three participants were currently receiving support from a physical therapist for physical activity. Support of a sports instructor was also mentioned.

And then I get a schedule with pictures and I have to do that for six weeks, and after six weeks we evaluate that and then I get something else or you continue to see if the pace is increased, things like that. So a bit under supervision.

One patient mentioned support of a GP by increasing physical activity. The doctor could give support by monitoring the blood pressure and the cholesterol level so that the patient remains more motivated to keep it up.

Maybe the doctor, … actually the doctor has to regularly measure blood pressure and cholesterol. So, we also have agreements for that. Maybe the doctor could mean something in that. I think so, I think it will matter to me. But for the time being I will get up every morning and get on my bike.

Support by a dietician for changing diet has been mentioned by one male participant in order to get some more guidelines for good cholesterol levels. One participant already had a dietician for, among other things, diet plans.

She finds schedules for me for food, for sports and for weight … I wanted to lose 10 kilos, because I had gained 10 kilos after that accident. And half of that is now reached, still five kilos .. and she will accompany me for a few months. And she always sends me these things [applications].

Social support has been mentioned in different ways. Some participants currently get emotional support to move more. For example, one patient receives support from his wife when walking and others receive encouragement from their partner to get more exercise.

Yes, I will cycle with her. Yes since now..

Patients also named the importance of support in quitting smoking and in insisting on reducing alcohol consumption.

Nothing. Yes, my daughters and son. They also say: not too much!

## 4 DISCUSSION

Patients seem unable to adequately appraise their own health‐related behavior. More than half of the patients were satisfied with their lifestyle and had no urgency to change. Self‐efficacy as coping factor was the most important determinant (both barrier and facilitator), and fear as threat factor was named as facilitator for health‐related behavior change by half of the patients. The majority of the patients did not need support or already received support in changing their lifestyle. Patients indicated knowledge, guidelines, and social support as most needed to change health‐related behavior and to preserve a healthy lifestyle.

Based on baseline characteristics, many of these patients seem not to have a healthy lifestyle (28% smokers, 22% alcohol abuse, and 47% overweight). Although the participants know what a healthy lifestyle constitutes, they seemed unable to assess their own lifestyle properly. However, we did not assess all actual health‐related behaviors (such as physical activity and diet) in this study. An earlier quantitative study showed that risk assessment and knowledge about risk factors is not optimal after ischemic stroke (Croquelois & Bogousslavsky, 2006). In our study, patients indicated knowledge and guidelines as facilitating factors for health‐related behavior change. Several previous quantitative studies showed that many stroke patients express a lack of understanding and desire for further knowledge about all aspects of stroke disease (Rodgers et al., 2001). In the protection motivation theory, coping and threat appraisal is influenced by sources of information, such as verbal contemplation, personal experiences, or observational learning. Earlier studies named this influence of the cognitive mediation process knowledge (Chamroonsawasdi et al., 2017). The need for guidelines can also be explained by the fact that the coping and threat appraisal is influenced by sources of information. Besides knowledge, social support has been indicated as most needed to change behavior. In line with this result, social support has been found as important factor for changing physical activity after stroke in many other quantitative and qualitative studies (Adeniyi et al., 2012; Barker & Brauer, 2005; Graham et al., 2008; Lennon, Doody, et al., 2013; Morris et al., 2012; Prout et al., 2016; Resnick et al., 2008). Low self‐efficacy appeared to be the strongest barrier for behavior change after TIA or ischemic stroke. Self‐efficacy has been found to have a direct effect on health‐related behavior and is the strongest predictor of health‐related behavior change (Schwarzer, 1995). Social support has no direct role in the protection motivation theory; however, self‐efficacy is influenced by social persuasion (Bandura, 1998; Marks et al., 2005). In our previous prospective cohort study, we found that self‐efficacy was the strongest determinant of intention to stop smoking, increase physical activity, and improve healthy diet (Brouwer‐Goossensen et al., 2016). Self‐efficacy was a powerful predictor of intention to change in other quantitative cardiovascular studies (Garcia & Mann, 2003; Sniehotta et al., 2005; Sol et al., 2005, 2006; Vries et al., 1988) . Therefore, self‐efficacy can be seen as a barrier and facilitator as patients in our study mentioned. Response efficacy and fear were also named as facilitating factors. In line with our results, earlier quantitative studies in cardiovascular and stroke patients showed response efficacy and fear as determinants of health behavior change (Blanchard et al., 2009; Tulloch et al., 2009). In our previous study, we showed that fear of a recurrent stroke is often present and leads to a motivation to make changes to promote patients' health in order to avoid a new stroke (Bendz, 2003; Carlsson et al., 2009; Horne et al., 2014; Martin et al., 2002; Nordin et al., 2015; Townend et al., 2006) In this study, fear of recurrence and response efficacy were also determinants of intention to change health behavior after TIA or ischemic stroke (Brouwer‐Goossensen et al., 2016). To the best of our knowledge, there are no other studies focusing on fear and response efficacy in relation to actual health behavior change in patients with TIA or ischemic stroke.

The results of our study show that patients after TIA or ischemic stroke often feel no urgency to change. Patients may not have enough knowledge to properly assess their lifestyle and severity of stroke recurrence. As mentioned before, this lack of knowledge can influence the coping and threat appraisal. Stroke patients are known to have a low awareness of risk factors for stroke. Although awareness is not part of the protection motivation theory, it is seen as a first step (before knowledge) toward acquiring skills and attitudes to change behavior (Carleton et al., (1996)). However, patients indicated to have sufficient knowledge and most patients indicated that they did not need support. When patients felt the need to change, they indicated knowledge as the most necessary factor for changing health‐related behaviors and felt the need to know more about guidelines. Several patients mentioned fear as a facilitator for health‐related behavior change. However, this fear will increase if patients have sufficient knowledge to estimate the severity. Besides knowledge, self‐efficacy appeared to play an important role. When patients are convinced of the importance of behavioral change and have enough knowledge of the guidelines, self‐confidence is needed to proceed to actual change. Response efficacy was also mentioned as a facilitator of health behavior change. It may be an important determinant as behavior change is hard to accomplish, and patients are only willing to change when they believe that making the change is effective in reducing the risk of other events. Lack of knowledge can also play a role in this determinant. If patients are not aware of the effects of health‐related behavior change, they will be less likely to change. In our earlier study, we found a gap between intention and actual change. Patients had the intention to change, and high self‐efficacy and fear were present, but there was no actual change. Possibly these patients did not know how to change their lifestyle, as patients in our present study mentioned knowledge and guidelines as the most needed factor to health‐ related behavior change and to preserve a healthy lifestyle. However, knowledge is not sufficient to adopt a healthy lifestyle, because other barriers to behavioral change often overrule the advice (Ellis et al., 2005; Vries et al., 1988). If there is sufficient knowledge but self‐efficacy is low, it will be difficult to proceed to actual change. Only two studies (one quantitative and one qualitative) focused on knowledge in relation to health behavior change in patients with ischemic stroke. Both studies found no difference in behavioral change in lifestyle (Allison et al., (2008); Blanchard et al., 2009). A meta‐analysis of 47 studies examining the relation between intention and health behaviors using a variety of populations showed that a medium‐to‐large change in intention led to only a small‐to‐medium change in behavior (Webb & Sheeran, 2006). This earlier mentioned “intention–behavior gap” strengthens the experience that changing complex behaviors such as physical inactivity requires more than simply the formation of good intentions (Schwarzer, 2008). To bridge this gap, the Health Action Process Approach uses an individualized and engaging action and coping planning component. Action planning means that it is important to make a detailed mental representation of “when,” “where,” and “how” an intended behavioral action has to be performed. Action control is a self‐regulatory process of *self‐monitoring* one's own behavior, *awareness* of the intended behavior and the *effort* one makes in performing the intended behavior (Sniehotta et al., 2005). Next to self‐efficacy, action planning and control is crucial to bridge the gap between intentions to change behavior and actual behavior change and behavior maintenance. Therefore, interventions focusing not only on increasing self‐efficacy and self‐management but also on action planning and action control can possibly bridge the intention behavior gap in these patients.

Strength of this study is that patients were interviewed at home in their own environment. Therefore, social desirability does not seem to play a large role in this study. Another strength is the qualitative aspect of this study. As far as we know patients' perspectives on determinants of health‐related behavior change after ischemic stroke have not been studied qualitatively before. Since many discussion points returned and were comparable between patients, saturation was reached, and the sample of 18 patients seems to have been taken properly. This study has also some limitations. Patients do not seem to adequately appraise their lifestyle. We cannot be sure how much this judgment differs from the actual lifestyle of the patients as we have not assessed their actual health‐related behavior. We should have assessed their actual lifestyle more thorough. When patients think they have a healthy lifestyle, a conversation about changing health behavior can be difficult. Therefore, it possibly would be better to use questionnaires to assess their lifestyle first. Another limitation is the use of semistructured interviews. Although the interview was as open as possible, sometimes the interviewer did a suggestion that gave the patient a direction, because some issues had to be addressed. On the other hand, some determinants, such s “perceived severity,” were still not discussed much. The short time between the ischemic stroke or TIA and the interview has advantages and limitations. On the one hand, patients just experienced their ischemic stroke or TIA and made decisions about their behavior change. On the other hand, some patients did not think about their health behavior yet at the time of the interview and were mainly focused on recovering.

In conclusion, this study suggests that patients with recent TIA or ischemic stroke often do not have a high intention to change health‐related behavior. Patients understand what constitutes a healthy lifestyle, but seem unable to adequately appraise their own health‐related behavior. In this study, more than half of the patients felt no urgency to change their lifestyle. Patients indicated that knowledge and guidelines for healthy lifestyle can help in changing health behavior. Possibly these findings are related as patients are not able to assess their lifestyle properly as knowledge of what a healthy lifestyle entails is missing. Increasing knowledge could therefore not immediately increase the motivation for change, but possibly increase the awareness of what a healthy lifestyle consists of and how the patient's current lifestyle relates to this. Active screening on lifestyle risk factors with questionnaires can possibly help in this process. Patients also indicated social support as most needed factor to change behavior. Increasing awareness among patients and their relatives could therefore be a first step toward change. In addition, self‐efficacy also appears to play a major role in changing the lifestyle in this study. Future studies should therefore focus on investigating opportunities to effectively increase knowledge, social support, and self‐efficacy to promote lifestyle change.

## CONFLICT OF INTEREST

All the authors report no disclosures.

## AUTHOR CONTRIBUTIONS

M.A. Mastenbroek‐ de Jong designed the study with J.E.W.C van Gemert‐Pijnen, E.Taal and M. den Hertog. M. Mastenbroek‐ de Jong interviewed, transcribed, and analyzed the interviews. D. Brouwer‐Goossensen and E.Taal reviewed this classification. M. Mastenbroek‐ de Jong wrote the first (Dutch) draft of the manuscript. D. Brouwer‐ Goossensen wrote the original manuscript and added tables and figures in consultation with E.Taal and H. den Hertog. All authors commented on the manuscript.

### Peer Review

The peer review history for this article is available at https://publons.com/publon/10.1002/brb3.1993.

## Data Availability

The data that support the findings of this study are available from the corresponding author upon reasonable request.
